# Excessive sulfur oxidation in endoplasmic reticulum drives an inflammatory reaction of chondrocytes in aging mice

**DOI:** 10.3389/fphar.2022.1058469

**Published:** 2022-10-24

**Authors:** Kun Chen, Xianzuo Zhang, Zhi Li, Xingshi Yuan, Daijie Fu, Kerong Wu, Xifu Shang, Zhe Ni

**Affiliations:** Department of Orthopedics, The First Affiliated Hospital of USTC, Division of Life Sciences and Medicine, University of Science and Technology of China, Hefei, China

**Keywords:** aging, chondrocyte, osteoarthritis, sulfur oxidation of endoplasmic reticulum, proteomics

## Abstract

Osteoarthritis, as a common joint disease among middle-aged and elderly people, has many problems, such as diverse pathogenesis, poor prognosis and high recurrence rate, which seriously affects patients’ physical and mental health and reduces their quality of life. At present, the pathogenesis of osteoarthritis is not completely clear, and the treatment plan is mainly to relieve symptoms and ensure basic quality of life. Therefore, it is particularly urgent to explore the pathogenesis of osteoarthritis. Protein, as organic macromolecule which plays a major role in life activities, plays an important role in the development of disease. Through protein omics, this study found that with the increase of age, excessive sulfur oxidation occurred in endoplasmic reticulum of chondrocytes, which then drove the occurrence of inflammatory reaction, and provided a direction for the follow-up molecular targeted.

## Introduction

Osteoarthritis (OA) is a common chronic degenerative joint disease, which is characterized by swelling, pain and stiffness of joints. In severe cases, it is accompanied by dysfunction and loss of mobility (Abdel-Rahman et al., 2020). Its main pathological damage is the loss and degeneration of articular cartilage (Jiang et al., 2022). At present, the specific pathogenesis of the disease is still unclear. Some factors can induce osteoarthritis, such as aging, obesity, inflammation, genetics, joint wear and tear, etc., Among them, aging is considered to be the main factor (Mrtens et al., 2022).

With the aging of the population in various countries, age-related diseases such as osteoarthritis are highly valued. According to the statistics of the World Health Organization, there are more than 350 million people with osteoarthritis worldwide, and one out of every six people in Asia suffers from osteoarthritis. Taking China as an example, as of 2017, the prevalence rate of knee arthritis was highest in the age group over 70 years old, with 26.3%, and the lowest in the age group of 15–39 years old, with 3.1% (Li et al., 2020). After the age of 40, the prevalence increased linearly with age. At present, there are many studies on aging and osteoarthritis, ranging from the RNA level to the protein level. Based on existing studies (Liu et al., 2021), (Miura et al., 2022)^,^ (Varela-Eirín et al., 2022), this study found that with the increase of age, the number of aging cells in articular cartilage increased, which promoted the occurrence of inflammatory aging-related secretory phenotype (SASP), and then led to an increase in the expression of inflammatory response-related genes and proteins. At the same time, it has been reported that osteoarthritis is related to bone metabolism. Osteoarthritis often leads to bone metabolism disorder, and the expression of genes involved in metabolism is decreased (Sebastian et al., 2020), (Eitner et al., 2022). At present, it is known that aging can lead to inflammation of joints and bone metabolism disorders, but the specific occurrence process and molecular regulation mechanism are still unclear, which could not provide a clear direction for disease treatment.

We used four-dimensional protein omics to analyze the difference of protein in the articular cartilage of 2-month and 20-month-old mice. Results showed that compared with the 2-month-old mice, the 20-month-old mice had enhanced immune response and decreased metabolic response, which was consistent with previous research results. Through protein omics analysis and Western Blot experiment, this study proved that the changes were caused by excessive sulfur oxidation in endoplasmic reticulum and increased folding of pseudoproteins after aging, which increased metabolic pressure and promoted inflammatory reaction, thus providing a new idea for the treatment of osteoarthritis.

## Method

### Sample collection of experiment

The whole animal experimental process complied with the animal ethics of the First Affiliated Hospital of University of Science and Technology of China. First of all, C57BL/6 mice aged 2 and 20 months raised normally were selected as the control group (2-month-old) and the experimental group (20-month-old) and sacrificed by neck-break. The articular cartilage was separated by artificial peeling and stored at −80°C for further analysis.

### Proteomic analysis

Liquid nitrogen was added into the sample, and it was completely ground into powder. Then, 4 times the volume of lysis buffer was added, and the sample was broken by ultrasonic wave. The supernatant was collected by centrifugation at 12,000 *g* for 10 min at 4°C, and the protein concentration was measured by BCA kit. An equal amount of protein was taken from each sample, and the volume was adjusted to be consistent with that of the lysis solution. DTT with a final concentration of 5 mM was added and denatured at 56°C for 30 min. Then IAA with a final concentration of 11 mM was added, and incubated in the dark at room temperature for 15 min. After centrifugation at room temperature of 12,000 g for 20 min, firstly, 8 M urea was used to replace urea three times, and then the replacement buffer was used to replace urea three times. Trypsin was added at the ratio of 1:50, and enzymolysis was carried out overnight. The peptide fragments were recovered by centrifugation at 12,000 *g* for 10 min at room temperature, and then recovered once with ultra-pure water, and the peptide fragment solutions were combined two times. The peptide fragment solution was further subjected to HPLC-MS.

### Western blot

After the tissue proteins were extracted from the protein, the concentration of protein was determined by BCA kit, and the load of each sample was adjusted according to the concentration of protein. After loading 12% acrylamide gel, run it at 80 v for 30 min. After the mark starts to separate, adjust the voltage to 120 v, and then run. When the loading buffer flows to the bottom of the separation gel, the membrane transfer begins. The order was three layers of filter paper, gel, PVDF membrane and three layers of filter paper. According to the molecular weight, the constant flow membrane time is controlled. After the membrane transfer, the protein bands were initially dyed with Ponceau S, and the desired target bands were cut. After the target bands were blocked at room temperature for 1 h on a 5% skim milk, the primary antibodies were incubated overnight at 4°C. The next day, the membrane was washed 5 times with TBST for 5 minutes each time, and the second antibody was incubated at room temperature. The membrane was washed by TBST for 5 times, each time for 5 min.

## Result

### Experimental design and proteomics results

**FIGURE 1:**
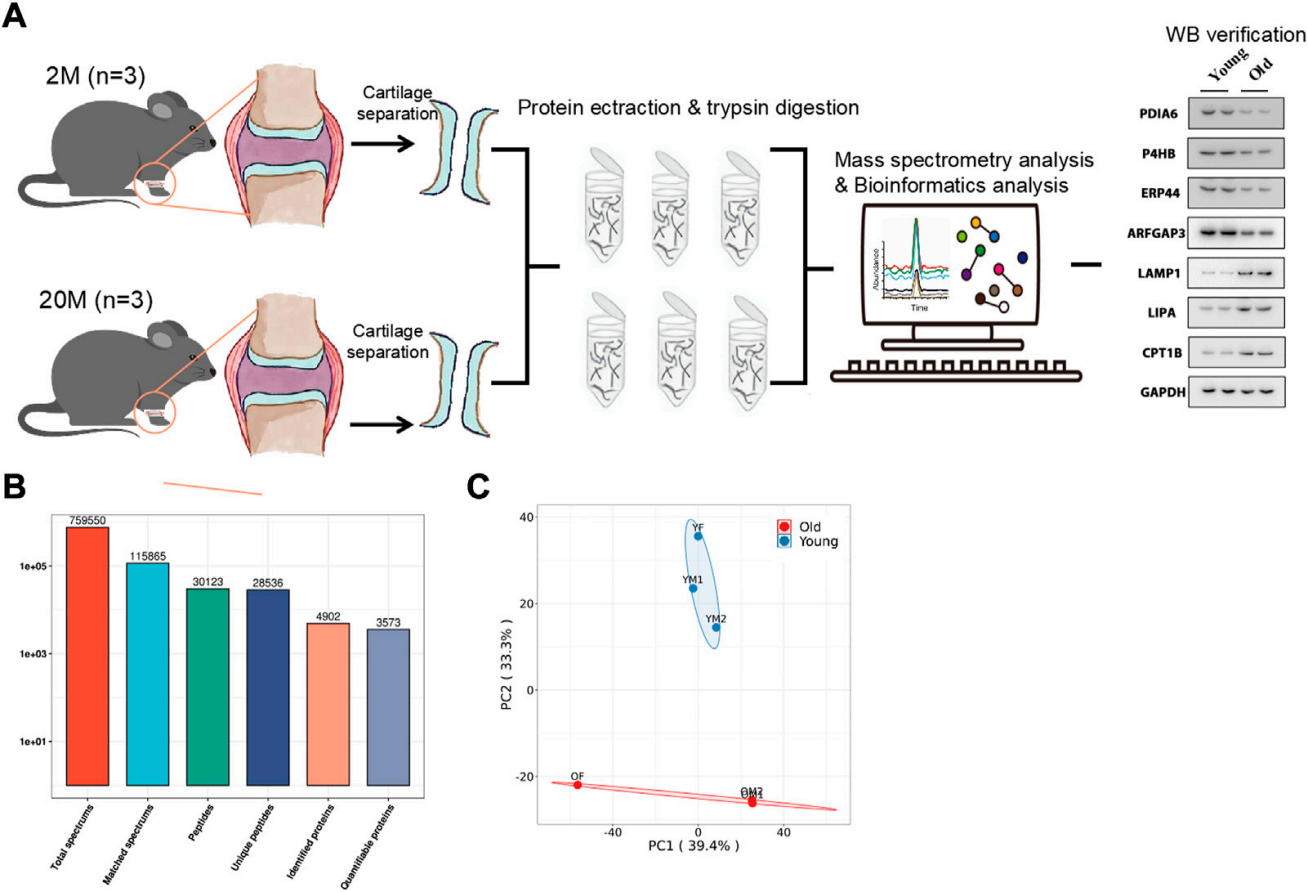
Overview of the proteomic analysis results. **(A)** The work flow of this paper. **(B)** Number of Peptides and proteins Identified by proteomics. **(C)** Principal Component Analysis Chart of Mouse articular cartilage.

As shown in Figure 1A, three mice aged 2 months (the young group) and three mice aged 20 months (the old group) were selected, and the articular cartilage was obtained by artificial stripping and subjected to Four-Dimensional Proteomics. Next, a total of 28,536 specific peptides and 4902 corresponding proteins fragments were detected by protein quantitative mass spectrometry (Figure 1B). The repeatability of quantitative analysis of protein was evaluated by principal component analysis (Figure 1C). These results showed that a group of obviously different protein of mouse articular cartilage were obtained for enrichment analysis.

### Unbiased bioinformatics analysis of articular cartilage in young and aging mice

**FIGURE 2:**
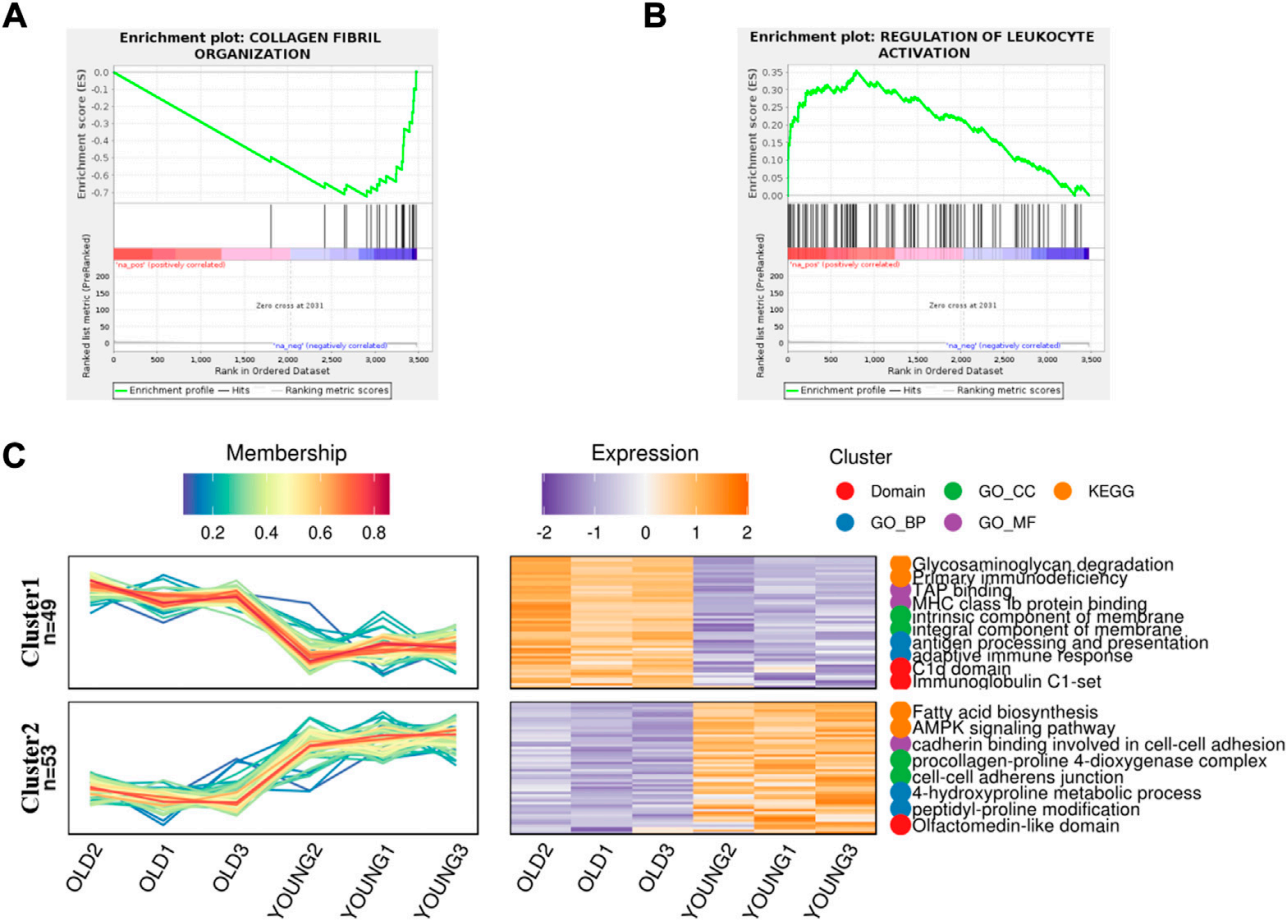
Gene Set Enrichment analysis and gene expression trend analysis in aging chondrocytes. **(A)** Gene set enrichment analysis of post-aging upregulated protein (*p* < 0.01). **(B)** Gene set enrichment analysis of post-aging downregulated protein (*p* < 0.05). **(C)** Trend analysis showed the related pathways involved in upregulating and downregulating proteins after aging.

In order to better understand the difference of biological function of articular cartilage between the young and the old group, firstly, gene set enrichment analysis was performed on all significantly expressed proteins. Compared with the control group, the expression of collagen fiber tissue-related proteins in the old group was generally downregulated (Figure 2A), while the expression proteins related to the regulation of leukocyte activation were generally upregulated in the old group (Figure 2B). This further shows that aging can lead to degeneration of articular cartilage and loss of collagen fibers, accompanied by cell inflammation. At present, many studies show that patients with metabolic syndrome are often accompanied by osteoarthritis (Afifi et al., 2018), (Orlenko et al., 2020), indicating that metabolic disorders is a possible cause of osteoarthritis. According to reports, there are some obstacles in glucose metabolism (Li et al., 2022), phospholipid metabolism (Zhai, 2021) and amino acid metabolism (Chen et al., 2018) in patients with osteoarthritis or animal models, which can provide ideas for the diagnosis and prevention of osteoarthritis. At the same time, proteomics also found that the expression of fatty acid biosynthesis related proteins in cartilage of OA aged mice was downregulated (Figure 2C), but the specific formation factors were still unclear. Therefore, statistical analysis was made on the significantly upregulated and downregulated proteins.

### Metabolic disorder caused by misfolding of endoplasmic reticulum protein

**FIGURE 3:**
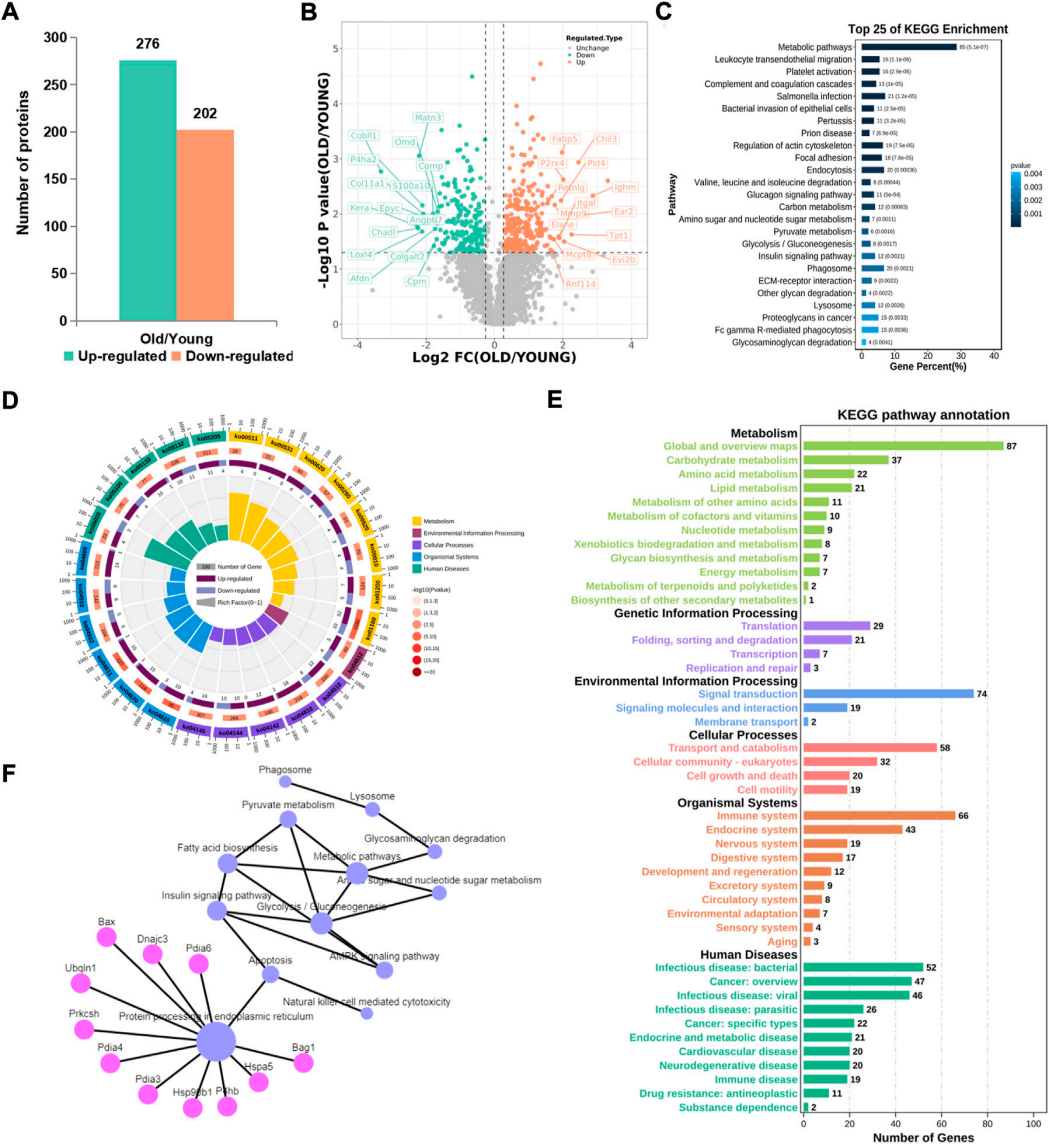
KEGG pathway analysis of proteins with significant difference. **(A)** Number of differential proteins in articular cartilage after aging (Ratio>1.2, *p* < 0.01). **(B)** Volcano plots showing differential proteins. **(C)** Top 25 KEGG pathways of differential proteins. **(D)** The radar chart showed the relationship between upregulated and downregulated genes and the KEGG pathway. **(E)** Sub-terms included in the radar chart term. **(F)** Network analysis of KEGG pathways.

Compared with the control group, 276 proteins were significantly up-regulated and 202 proteins were significantly downregulated in the old group (Figure 3A). From the volcano map, we can clearly see the proteins with significant difference in expression (Figure 3B). By summarizing the subcellular localization of the upregulated and downregulated proteins, no significant differences in protein localization were observed (Supplementary Figure S1). The top 25 KEGG pathways were sequenced and enriched by differentially expressed proteins. The most significant enrichment pathway was the change in metabolic pathway, and phagocytes, lysosomes and other signaling pathways were enriched (Figure 3C). Based on radar chart analysis of enrichment KEGG pathway, it was found that the functions of enrichment pathway are mainly metabolism, environmental treatment and cell processing (Figure 3D). Among them, changes of metabolic pathways are unique to some carbohydrate metabolism, amino acid metabolism and lipid metabolism. At the same time, it is also found that protein translation, protein folding storage and degradation pathways in the gene information processing are significantly abundant (Figure 3E). In order to clarify the relationship between these altered KEGG pathways, network diagram analysis was carried out, and it was found that endoplasmic reticulum protein processing played a leading role, and could cause a series of subsequent changes, such as apoptosis, fatty acid biosynthesis, phagocytosis, lysosomes, etc., (Figure 3F), which pointed out the direction for this study.

### Endoplasmic reticulum oxidative stress induces osteoarthritis

**FIGURE 4:**
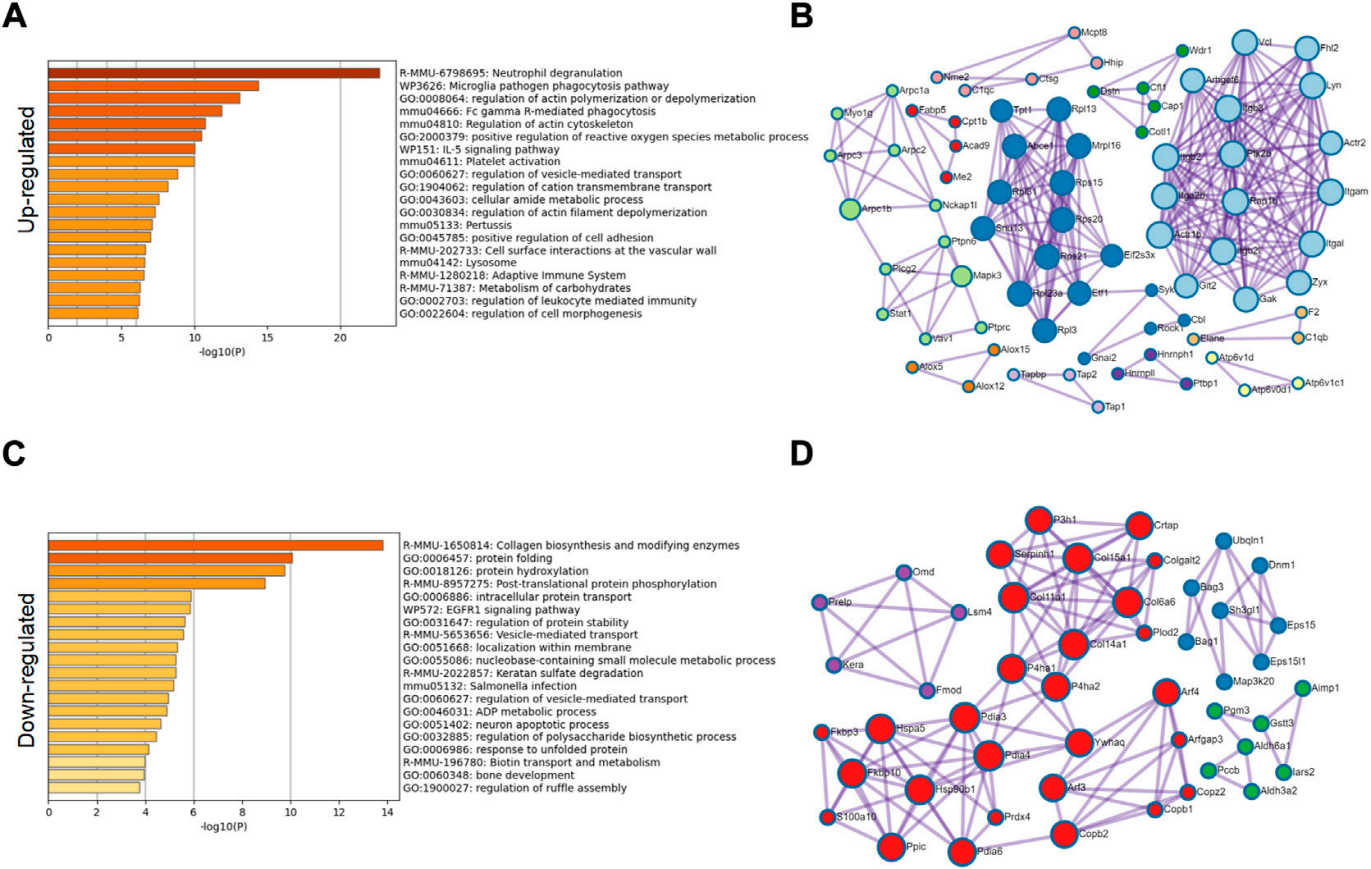
GO classification of significantly different proteins. **(A)** GO classification of 276 significantly upregulated proteins. **(B)** Network diagram analysis of upregulated proteins. **(C)** GO classification of 202 significantly downregulated proteins. **(D)** Network diagram analysis of downregulated proteins.

From a single level, GO analysis and network map analysis were used to analyze the significantly upregulated and downregulated protein, and the changes between them were observed. Among the upregulated proteins, GO analysis focuses on lysosomes, inflammatory immune response, carbohydrate metabolism and reactive oxygen species metabolism (Figures 4A,B). The main functional changes of the downregulated proteins were protein folding, intracellular protein stabilization, membrane localization, and error protein handling (Figures 4C,D). The main organelle responsible for translation and folding of protein is endoplasmic reticulum (Supplementary Figure S2A), indicating that endoplasmic reticulum played an important role in osteoarthritis. It has been reported that endoplasmic reticulum oxidative stress occurs in articular chondrocytes of OA patients, which promotes apoptosis of chondrocytes (Bian et al., 2018), (Cao et al., 2018). After treatment with antioxidants, they can protect the chondrocytes, thus alleviating disease (Fu et al., 2022), (Hecht et al., 2021), (Lin et al., 2021). Therefore, we conducted a further study on the causes of endoplasmic reticulum stress.

### Excessive sulfur oxidation in endoplasmic reticulum leads to protein misfolding reaction, inducing osteoarthritis

**FIGURE 5:**
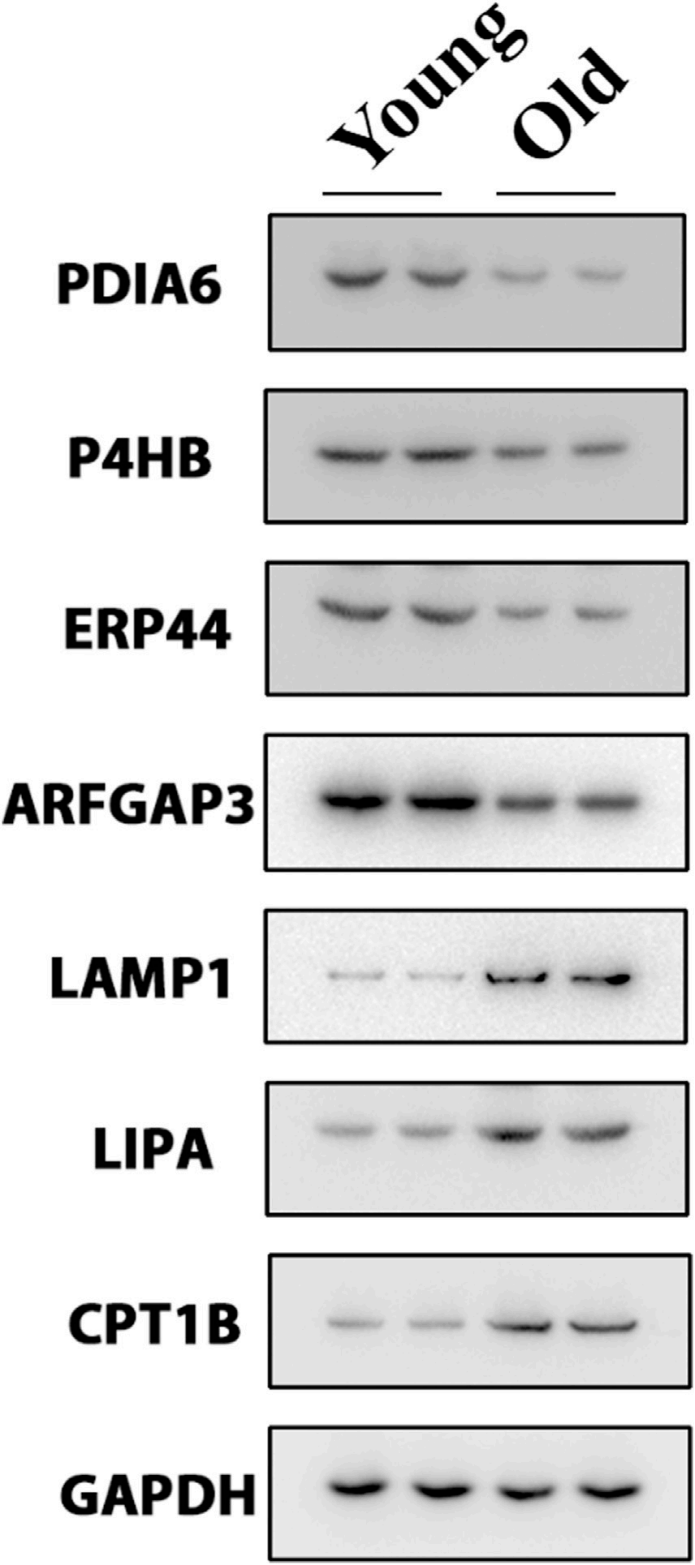
Western blot experiment verified the key differentially expressed proteins.

We selected key target proteins from the results of enrichment analysis (Supplementary Table S1). The results of the proteomics were verified by Western blot. Some disulfide isomerases located on the endoplasmic reticulum, such as PDIA6 (Choi et al., 2020), P4HB (Jang et al., 2019) and ERP44 (Liyanage et al., 2019), are thiol redox partners of thioredoxin family, assisting the folding of nascent protein and the production of disulfide bonds. The expression levels of these proteins in the chondrocytes of the old group decreased (Figure 5), indicating that aging would lead to the decline of the body’s ability to resist sulfur oxidation reaction, excessive oxidation and dysfunction of endoplasmic reticulum, and start the misfolding of protein, namely unfolded protein reaction (UPR). Meanwhile, the expression level of ARFGAP3 (a regulatory molecule responsible for intracellular material transport) decreased significantly in the old group (Figure 5), indicating that the vesicle transport system in the cell has failed, and the cell has produced a large amount of waste. This required the lysosome to initiate the self-clearance mechanism (Supplementary Figure S2B), remove the excess waste, and increase the expression levels of lysosomal related reaction enzymes LAMP1 (Chaudhry et al., 2022) and LIPA (Morris et al., 2017) (Figure 5). However, the implementation of these reactions requires a great deal of energy, which requires the decomposition of fatty acids to provide energy. Therefore, the expression of rate-limiting enzyme CPT1B in fatty acid oxidation increased (Figure 5).

However, osteoarthritis can cause disorder of fatty acid metabolism in articular chondrocytes (Figure 2C), and it can’t provide energy well, which leads to the untimely removal of excess waste, further aggravates the sulfur-oxygen reduction reaction of endoplasmic reticulum and the misfolding of protein, resulting in a vicious circle.

## Discussion

Osteoarthritis is an age-related disease with articular cartilage degeneration driven by multiple pathogenic factors. Cell senescence caused by age is the main pathogenic factor of osteoarthritis. Currently, the possible pathogenesis is mainly focused on endoplasmic reticulum stress of chondrocytes (Cao et al., 2018). In addition, many studies have found that some traditional Chinese medicine extracts can alleviate the pain of osteoarthritis patients by reducing the endoplasmic reticulum stress of chondrocytes (Fu et al., 2022)^,^ (Bian et al., 2018), but they still have not achieved the goal of targeted therapy.

In this study, proteomics was used to analyze the difference of protein expression in articular chondrocytes of 2-month-old and 20-month-old mice, and more than 400 different proteins were screened out. It has been found that the processing error of endoplasmic reticulum protein was the cause of cartilage metabolic dysfunction and inflammatory reaction. The experimental verification of related functional proteins showed that with the increase of age, the expression level of thioredoxin in endoplasmic reticulum decreased, and the oxidative stress of endoplasmic reticulum caused unfolded protein reaction (UPR) (Liang et al., 2021) and vesicle transport problem of protein folding errors, which led to excessive cell waste. A large number of lysosomes needed to be degraded, and more energy was needed to help the reaction. Therefore, the oxidative decomposition of fatty acids provides energy but also aggravates the oxidative stress response of cells (Zhang et al., 2022), forming a vicious circle. A large number of articles show that excessive oxidation and UPR can cause inflammatory reactions (Feng et al., 2021).

Here, we provide a new point of view, OA is a metabolic disease, and excessive sulfur oxidation in the endoplasmic reticulum leads to unfolded protein reaction, which then leads to inflammation. This provides a new direction for the symptomatic treatment of OA and also explains the relationship between ER stress and OA. However, the relevant mechanism in this research is not enough, it only provides basic research. In the current study, we only compared the samples from yang and old mice chondrocytes. It would be better also checking the expression of these different expressed proteins between normal and OA chondrocytes in mice and human. That is exactly what we will do in our later study. Also, there was a lack of research on molecular targeted therapy before, and the related research needed to be improved in the later stage.

## Data Availability

The datasets presented in this study can be found in online repositories. The names of the repository/repositories and accession numbers can be found below: http://www.proteomexchange.org/, PXD037302.

## References

[B1] Abdel-Rahman R. F. Abd-Elsalam R. M. Amer M. S. El-Desoky A. M. Mohamed S. O. (2020). Manjarix attenuated pain and joint swelling in a rat model of monosodium iodoacetate-induced osteoarthritis. Food Funct. 11 (1), 7960–7972. 10.1039/d0fo01297a 32839804

[B2] Afifi A. E. A. Shaat R. M. Gharbia O. M. Boghdadi Y. E. Eshmawy M. M. E. El-Emam O. A. (2018). Osteoarthritis of knee joint in metabolic syndrome. Clin. Rheumatol. 37 (10), 2855–2861. 10.1007/s10067-018-4201-4 30039268

[B3] Bian Y. Wang H. Sun S. (2018). Taurine alleviates endoplasmic reticulum stress in the chondrocytes from patients with osteoarthritis. Redox Rep. 23 (1), 118–124. 10.1080/13510002.2018.1445581 29494284 PMC6748701

[B4] Cao J. Zhang Y. Wang T. Li B. (2018). Endoplasmic reticulum stress is involved in baicalin protection on chondrocytes from patients with osteoarthritis. Dose. Response. 16 (4), 1559325818810636. 10.1177/1559325818810636 30505248 PMC6256307

[B5] Chaudhry N. Sica M. Surabhi S. Hernandez D. S. Mesquita A. Selimovic A. Riaz A. Lescat L. Bai H. Macintosh G. C. Jenny A. (2022). Lamp1 mediates lipid transport, but is dispensable for autophagy in Drosophila. Autophagy 18, 2443–2458. 10.1080/15548627.2022.2038999 35266854 PMC9542896

[B6] Chen R. Han S. Liu X. Wang K. Zhou Y. Yang C. Zhang X. (2018). Perturbations in amino acids and metabolic pathways in osteoarthritis patients determined by targeted metabolomics analysis. J. Chromatogr. B Anal. Technol. Biomed. Life Sci. 1085, 54–62. 10.1016/j.jchromb.2018.03.047 29631251

[B7] Choi J. H. Zhong X. Zhang Z. Su L. McAlpine W. Misawa T. Liao T. C. Zhan X. Russell J. Ludwig S. Li X. (2020). Essential cell-extrinsic requirement for PDIA6 in lymphoid and myeloid development. J. Exp. Med. 217 (4), e20190006. 10.1084/jem.20190006 31985756 PMC7144532

[B8] Eitner A. Sparing S. Kohler F. C. Muller S. Hofmann G. O. Kamradt T. Schaible H. G. Aurich M. (2022). Osteoarthritis-Induced metabolic alterations of human hip chondrocytes. Biomedicines 10 (6), 1349. 10.3390/biomedicines10061349 35740371 PMC9220245

[B9] Feng Z. Z. Luo N. Liu Y. Hu J. N. Ma T. Yao Y. M. (2021). ER stress and its PERK branch enhance TCR-induced activation in regulatory T cells. Biochem. Biophys. Res. Commun. 563, 8–14. 10.1016/j.bbrc.2021.05.061 34058476

[B10] Fu C. Qiu Z. Huang Y. Lin Q. Jin L. Tu H. Ye J. Zheng C. Zhong W. Ma D. (2022). Achyranthes bidentata polysaccharides alleviate endoplasmic reticulum stress in osteoarthritis via lncRNA NEAT1/miR-377-3p pathway. Biomed. Pharmacother. 154, 113551. 10.1016/j.biopha.2022.113551 35988424

[B11] Hecht J. T. Veerisetty A. C. Wu J. Coustry F. Hossain M. G. Chiu F. Gannon F. H. Posey K. L. (2021). Primary osteoarthritis early joint degeneration induced by endoplasmic reticulum stress is mitigated by resveratrol. Am. J. Pathol. 191 (9), 1624–1637. 10.1016/j.ajpath.2021.05.016 34116024 PMC8420863

[B12] Jang I. Pottekat A. Poothong J. Yong J. Lagunas-Acosta J. Charbono A. Chen Z. Scheuner D. L. Liu M. Itkin-Ansari P. Arvan P. (2019). PDIA1/P4HB is required for efficient proinsulin maturation and ß cell health in response to diet induced obesity. Elife 8, e44528. 10.7554/eLife.44528 31184304 PMC6559792

[B13] Jiang S. Zhang C. Lu Y. Yuan F. (2022). The molecular mechanism research of cartilage calcification induced by osteoarthritis. Bioengineered 13 (5), 13082–13088. 10.1080/21655979.2022.2078025 35611765 PMC9276012

[B14] Li D. Li S. Chen Q. Xie X. (2020). The prevalence of symptomatic knee osteoarthritis in relation to age, sex, area, region, and body mass index in China: A systematic review and meta-analysis. Front. Med. 7, 304. 10.3389/fmed.2020.00304 PMC7378378 32766258

[B15] Li K. Ji X. Seeley R. Lee W. C. Shi Y. Song F. Liao X. Song C. Huang X. (2022). Impaired glucose metabolism underlies articular cartilage degeneration in osteoarthritis. FASEB J. 36 (6), e22377. 10.1096/fj.202200485R 35608871

[B16] Liang W. Qi W. Geng Y. Wang L. Zhao J. Zhu K. (2021). Necroptosis activates UPR sensors without disrupting their binding with GRP78. Proc. Natl. Acad. Sci. U. S. A. 118 (39), e2110476118. 10.1073/pnas.2110476118 34544877 PMC8488584

[B17] Lin Z. Teng C. Ni L. Zhang Z. Lu X. Lou J., (2021). Echinacoside upregulates Sirt1 to suppress endoplasmic reticulum stress and inhibit extracellular matrix degradation *in vitro* and ameliorates osteoarthritis *in vivo* . Oxid. Med. Cell. Longev. 2021, 3137066. 10.1155/2021/3137066 34777682 PMC8580641

[B18] Liu W. Brodsky A. S. Feng M. Liu Y. Ding J. Jayasuriya C. T., (2021). Senescent tissue-resident mesenchymal stromal cells are an internal source of inflammation in human osteoarthritic cartilage. Front. Cell Dev. Biol. 9, 725071. 10.3389/fcell.2021.725071 34552931 PMC8450518

[B19] Liyanage D. S. Omeka W. K. M. Lee J. (2019). Molecular characterization, host defense mechanisms, and functional analysis of ERp44 from big-belly seahorse: A novel member of the teleost thioredoxin family present in the endoplasmic reticulum. Comp. Biochem. Physiol. B Biochem. Mol. Biol. 232, 31–41. 10.1016/j.cbpb.2019.02.006 30797055

[B20] Ma J. Chen T. Wu S. Yang C. Bai M. Shu K., (2019). iProX: an integrated proteome resource. Nucleic Acids Res. 47 (D1), D1211–D1217. 10.1093/nar/gky869 30252093 PMC6323926

[B21] Miura Y. Endo K. Komori K. Sekiya I. (2022). Clearance of senescent cells with ABT-263 improves biological functions of synovial mesenchymal stem cells from osteoarthritis patients. Stem Cell Res. Ther. 13 (1), 222. 10.1186/s13287-022-02901-4 35658936 PMC9166575

[B22] Morris G. E. Braund P. S. Moore J. S. Samani N. J. Codd V. Webb T. R. (2017). Coronary artery disease-associated LIPA coding variant rs1051338 reduces lysosomal acid lipase levels and activity in lysosomes. Arterioscler. Thromb. Vasc. Biol. 37 (6), 1050–1057. 10.1161/ATVBAHA.116.308734 28279971 PMC5444428

[B23] Mrtens N. Mrz V. Bertrand J. Lohmann C. H. Berth A (2022). Radiological changes in shoulder osteoarthritis and pain sensation correlate with patients' age. J. Orthop. Surg. Res. 17 (1), 1–9. 10.1186/s13018-022-03137-x 35570309 PMC9107673

[B24] Orlenko V. Tronko M. Bolgarskaya S. Yelizarova O. (2020). Georgian Med. News 301, 98–105. 32535572

[B25] Sebastian A. Murugesh D. K. Mendez M. E. Hum N. R. Rios-Arce N. D. McCool J. L., (2020). Global gene expression analysis identifies age-related differences in knee joint transcriptome during the development of post-traumatic osteoarthritis in mice. Int. J. Mol. Sci. 21 (1), 364. 10.3390/ijms21010364 PMC6982134 31935848

[B26] Varela-Eirín M. Carpintero-Fernández P. Guitián-Caamaño A. Varela-Vazquez A. Garcia-Yuste A. Sanchez-Temprano A., (2022). Extracellular vesicles enriched in connexin 43 promote a senescent phenotype in bone and synovial cells contributing to osteoarthritis progression. Cell Death Dis. 13 (8), 681. 10.1038/s41419-022-05089-w 35931686 PMC9355945

[B27] Zhai G. (2021). Clinical relevance of biochemical and metabolic changes in osteoarthritis. Adv. Clin. Chem. 101, 95–120. 10.1016/bs.acc.2020.06.001 33706891

[B28] Zhang H. Lu L. Zhao C. Liu Q. Zhou Q. Zhang Y., (2022). Lipid metabolism disorders contribute to hepatotoxicity of ICR mice induced by nitrosamines exposure. Environ. Int. 167, 107423. 10.1016/j.envint.2022.107423 35908391

