# Supplementary material for: Excessive sulfur oxidation in endoplasmic reticulum drives an inflammatory reaction of chondrocytes in aging mice
Source: Front Pharmacol. 2022 Oct 24;13:1058469. doi: 10.3389/fphar.2022.1058469 (PMC9638109; doi:10.3389/fphar.2022.1058469)
Supplement: Supplementary file 1 [file Table1.DOCX]

Supplementary Material


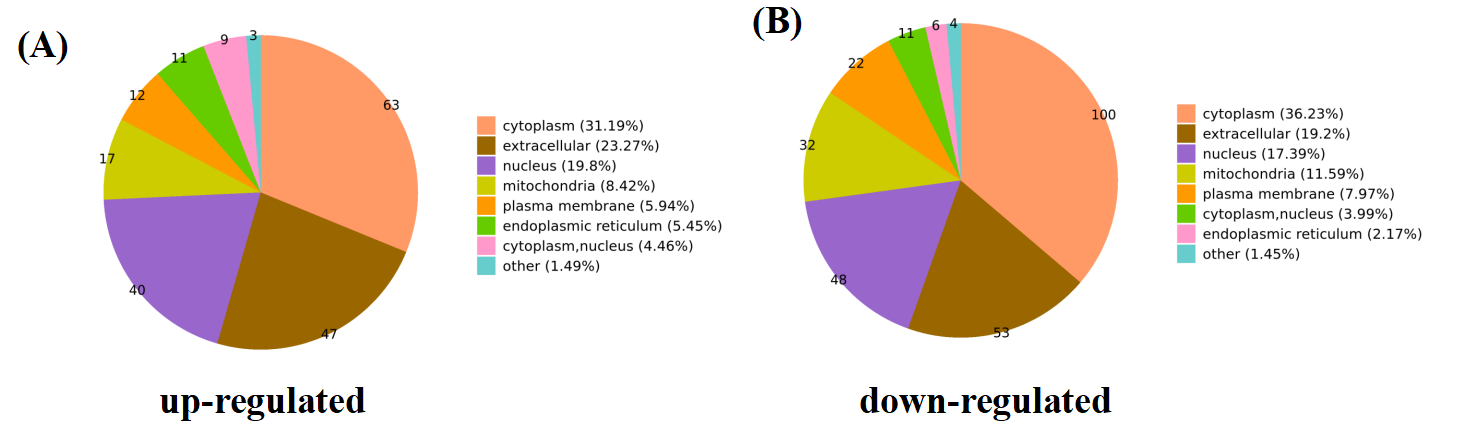


**Supplementary Figure 1.** **Subcellular localization statistics of significantly different proteins.**(A) Subcellular localization statistics of significantly up-regulated proteins. (B) Subcellular localization statistics of significantly down-regulated proteins.

| **Protein accession** | **Gene name** | **Old/Young Ratio** | **Old/Young P value** | **Regulated Type** |
| --- | --- | --- | --- | --- |
| **Q922R8** | **PDIA6** | **0.6248** | **0.042049059** | **Down** |
| **P09103** | **P4HB** | **0.4981** | **0.000682968** | **Down** |
| **Q9D1Q6** | **ERP44** | **0.6301** | **0.033157706** | **Down** |
| **Q9D8S3** | **ARFGAP3** | **0.531** | **0.040743877** | **Down** |
| **P11438** | **LAMP1** | **1.7884** | **0.003832314** | **Up** |
| **Q9Z0M5** | **LIPA** | **2.2407** | **0.002850983** | **Up** |
| **Q924X2** | **CPT1B** | **2.1516** | **0.000175367** | **Up** |

**Supplementary Table 1. List of differential proteins important for screening signal pathways.**

**
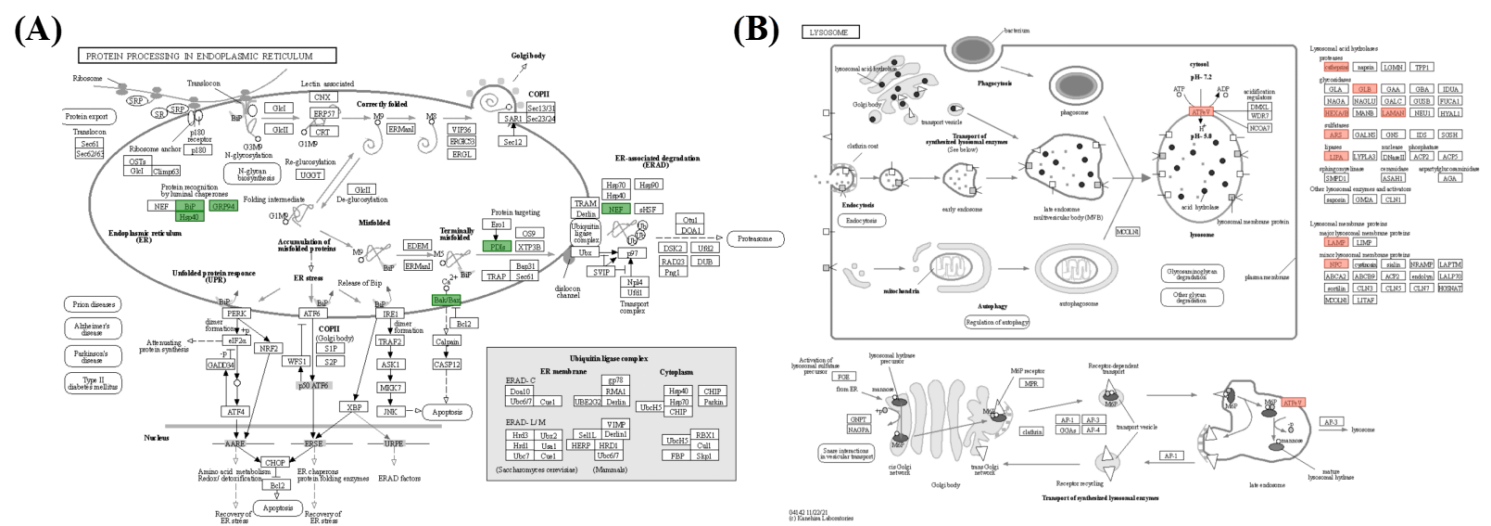
**

**Supplementary Figure 2.** **Mechanism path diagram. (**A) Mechanism diagram of unfolded protein reaction, the green-labeled protein indicated a protein with decreased expression in articular cartilage of aging mice. (B) Mechanism diagram for lysosomal clearance, the red-labeled protein indicated a protein with increased expression in articular cartilage of aging mice.
